# Knocking out the Vitamin D Receptor Enhances Malignancy and Decreases Responsiveness to Vitamin D3 Hydroxyderivatives in Human Melanoma Cells

**DOI:** 10.3390/cancers13133111

**Published:** 2021-06-22

**Authors:** Ewa Podgorska, Tae-Kang Kim, Zorica Janjetovic, Krystyna Urbanska, Robert C. Tuckey, Sejong Bae, Andrzej T. Slominski

**Affiliations:** 1 Department of Dermatology, University of Alabama at Birmingham, Birmingham, AL 35294, USA; epodgorska@uabmc.edu (E.P.); tkim@uabmc.edu (T.-K.K.); zjanjetovic@uabmc.edu (Z.J.); 2 Department of Biophysics, Faculty of Biochemistry, Biophysics and Biotechnology, Jagiellonian University in Kraków, 31-007 Kraków, Poland; krystyna.urbanska@uj.edu.pl; 3 School of Molecular Sciences, University of Western Australia, Perth, WA 6009, Australia; robert.tuckey@uwa.edu.au; 4 Department of Medicine, University of Alabama at Birmingham, Birmingham, AL 35294, USA; sbae@uabmc.edu; 5 O’Neal Comprehensive Cancer Center, University of Alabama at Birmingham, Birmingham, AL 35294, USA; 6 VA Medical Center, Birmingham, AL 35294, USA

**Keywords:** melanoma, vitamin D, vitamin D receptor, active forms of vitamin D, malignancy

## Abstract

**Simple Summary:**

Active forms of vitamin D3, including 1,25(OH)_2_D3, 20(OH)D3 and 1,20(OH)_2_D3, inhibited cell proliferation, migration rate and the ability to form colonies and spheroids in the wild-type melanoma cell line, while cells with the vitamin D receptor (VDR) silenced showed an increased but not complete resistance to their action. Furthermore, silencing of the VDR in melanoma cells enhanced their proliferation as well as spheroid and colony formation and increased their migration rate. Previous clinicopathological studies have shown an inverse correlation between VDR expression, melanoma progression and poor outcome of the disease. Thus, the expression of VDR is not only necessary for the inhibition of melanoma growth by active forms of vitamin D, but the VDR can also function as a melanoma tumor suppressor gene.

**Abstract:**

Vitamin D3 is not only involved in calcium and phosphate metabolism in humans, but it can also affect proliferation and differentiation of normal and cancer cells, including melanoma. The mechanism of the anti-cancer action of vitamin D3 is not fully understood. The nuclear vitamin D receptor (VDR) is crucial for the phenotypic effects of vitamin D hydroxyderivatives. VDR expression shows an inverse correlation with melanoma progression and poor outcome of the disease. In this study we knocked out the VDR in a human melanoma cell line using CRISPR methodology. This enhanced the proliferation of melanoma cells grown in monolayer culture, spheroids or colonies and their migration. Activated forms of vitamin D, including classical 1,25(OH)_2_D3, 20(OH)D3 and 1,20(OH)_2_D3, inhibited cell proliferation, migration rate and the ability to form colonies and spheroids in the wild-type melanoma cell line, while VDR KO cells showed a degree of resistance to their action. These results indicate that expression of VDR is important for the inhibition of melanoma growth induced by activated forms of vitamin D. In conclusion, based on our previous clinicopathological analyses and the current study, we suggest that the VDR can function as a melanoma tumor suppressor gene.

## 1. Introduction

Vitamin D3 (D3) is produced in the skin in two steps. Initially there is a photochemical reaction caused by the action of UVB radiation (290–315 nm) on 7-dehydrocholesterol (7DHC) in which the B ring is broken producing pre-vitamin D3 [1,2]. In the second reaction, vitamin D3 is formed from pre-vitamin D3 by its thermal isomerization at 37 °C over several hours [3]. Both the UVB intensity and the level of skin pigmentation affect the rate of vitamin D3 production [4]. Vitamin D3 is a fat-soluble prohormonal secosteroid that has endocrine, paracrine and autocrine functions [5]. Melanin absorbs UVB limiting the production of D3, and the same effect is achieved with clothing and sunscreen [6,7,8]. Skin, more specifically the epidermis, has the full capacity to produce and activate vitamin D3 [9,10,11].

The liver and kidneys are the main organs in which two-step activation of vitamin D3 occurs [1]. In the liver, vitamin D3 is metabolized by a vitamin D 25-hydroxylase (CYP2R1 or CYP27A1) to 25-hydroxyvitamin D3 (25(OH)D3), which is the main form of vitamin D in serum. 25(OH)D3 is further metabolized by 1α-hydroxylase (CYP27B1), mainly in the kidney proximal tubule, to 1α,25-dihydroxyvitamin D3 (1α,25(OH)_2_D3), the major hormonally active form of vitamin D3 [8]. 1α,25(OH)_2_D3 is transported via vitamin D binding protein (VDBP) in the bloodstream to target tissues such as the intestine, bones and kidneys where it regulates calcium and phosphate absorption and reabsorption, respectively. The concentration of 1α,25(OH)_2_D3 in the bloodstream regulates the expression of the inactivating enzyme, 25(OH)D3 24-hydroxylase (CYP24A1), which is induced when concentrations are high [12,13,14]. In addition to these classical pathways for vitamin D3 activation and inactivation, alternative metabolic pathways of vitamin D3 activation in the skin, including by keratinocytes [15] and dermal fibroblasts [16], are initiated by CYP11A1. Like 1,25(OH)_2_D3, the products of these pathways display anti-proliferative and differentiating abilities [3,17,18]. CYP11A1 is well known for catalyzing the hydroxylation of cholesterol at C22 and C20, followed by cleavage of the bond between C20 and C22 to generate pregnenolone, a common precursor for steroid hormones [19]. As well as the gonads and adrenal cortex, CYP11A1 is expressed in peripheral tissues such as the gastrointestinal tract, nervous system, immune system and skin [20,21]. It has more recently emerged that it is a vitamin D metabolizing enzyme with vitamin D serving as an alternative substrate to cholesterol [22,23]. The main metabolites of vitamin D that are formed by a single hydroxylation by CYP11A1 are 20(OH)D3, 22(OH)D3 and 17(OH)D3. These metabolites can be further hydroxylated by CYP11A1 to form 20,23(OH)_2_D3, 20,22(OH)_2_D3, 17,20(OH)_2_D3 and 17,20,23(OH)_3_D3. Moreover, the major product of this pathway, 20(OH)D3, may also serve as a substrate for CYP27A1, CYP24A1, CYP2R1 and CYP3A4 with hydroxylation occurring at C24, C25 or C26, while CYP27B1 hydroxylates most of these products at C-1α to produce the corresponding trihydroxyvitamin D metabolites. Overall, it has been estimated that this alternative metabolic pathway can produce more than 21 vitamin D hydroxyl-metabolites [24,25].

In target tissues, 1,25(OH)_2_D3 binds to the vitamin D receptor (VDR), a member of the nuclear receptor family, which includes ligand-activated transcription factors, and results in both genomic and non-genomic regulation of a variety of biological pathways [26,27,28]. The VDR is expressed in almost all tissues and cells, including the skin. Colston et al. [29] provided one of the first reports describing the presence of VDR outside of organs involved in calcium and phosphate homeostasis (intestines, kidneys and bone tissues), showing that the receptor is expressed in skin cell lines and in melanomas (malignant tumors originating from melanocytes) [30] and has anti-cancer properties. Subsequent experiments using human melanoma cell lines confirmed that the VDR is present in melanoma cells, although its expression level was heterogeneous between different cell lines [29]. The strongest expression of VDR was observed in normal skin, which decreased during progression of melanocytic lesions and during melanoma development. The VDR expression in perilesional skin was also significantly reduced in comparison to normal skin. Expression of VDR in various tumor tissues may suggest that it has an effect on tumorigenesis [31,32], for example in breast cancer [33] and lung cancer, and it might be related to the sex of patients [34]. The higher expression of VDR is also correlated with upregulated pathways that mediate the antitumor immunity and with downregulation of proliferative pathways [35].

Major types of skin cancer are basal and squamous cell carcinomas with melanoma being the most deadly skin neoplasm. The relationship between vitamin D and skin cancer is still under investigation. Vitamin D and novel vitamin D derivatives exhibit anti-proliferative activities on different skin cells, including melanoma cells [36,37,38,39,40,41]. Studies showed that vitamin D has a protective effect for patients with melanoma [42,43,44]. In the skin, 1,25(OH)_2_D3 plays an important role in regulating the epidermal barrier function and in regulating the growth and cycle of the hair follicles and also has anti-cancer, anti-proliferative and anti-inflammatory effects. It has recently been confirmed that it can inhibit skin cell death and DNA damage induced by UVR exposure. Due to its calcemic toxicity, the pharmacological use of 1,25(OH)_2_D3 is limited [45].

In 2010, Brożyna and co-authors showed that the level of tumor malignancy inversely correlates with VDR expression [31]. The strongest expression of VDR was observed in normal skin, and its expression decreased from normal skin through melanocytic nevi and melanoma to metastases. VDR expression in skin around moles and melanoma was also significantly reduced compared to normal skin [31], suggesting that it may serve as a marker of tumor progression [46,47]. Similar results were reported for breast cancer cells where VDR expression was inversely correlated to cancer malignancy [48], also seen in colon cancer [49]. In the current study we examined the effect of knocking out the VDR on melanoma malignant behavior.

## 2. Materials and Methods

### 2.1. Sources of Vitamin D3 Compounds

Both 1,25(OH)_2_D3 and 25(OH)D3 were purchased from Sigma. 1,20(OH)_2_D3 and 20(OH)D3 were enzymatically synthesized as described previously [50,51].

### 2.2. Culture of Melanoma Cells

WM164, a human melanoma line, was a gift from Dr M. Herlyn (Wistar Institute, Philadelphia, PA, USA). The cells were cultured using DMEM media supplemented with 10% serum (fetal bovine serum while growing cells and charcoal-treated serum during incubation with vitamin D compounds), 1% antibiotics and 5 µg/mL insulin (Sigma-Aldrich, St. Louis, MO, USA). Cells were cultured in 75 cm^2^ plastic bottles (TPP–Techno Plastic Products AG, Trasadingen, Switzerland) with a filter in an incubator at 37 °C with 5% CO_2_.

### 2.3. CRISPR/Cas Knock out of the VDR

**Figure 1:**
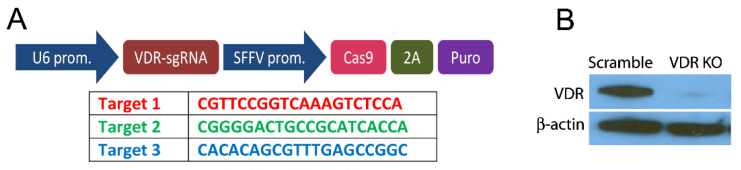
VDR knockout using CRISPR technology. (**A**) Main part of construct for generating sgRNA (top) and target sequence for VDR knockout (bottom). The lentivirus was produced by Applied Biological Materials Inc. (Richmond, BC, Canada) using pLenti-U6-sgRNA-SFFV-Cas9-2A-Puro. (**B**) Western blot of the VDR in scramble and VDR KO cells using β-actin as a loading control.

The clustered regularly interspaced short palindromic repeats (CRISPR) genetic engineering method was carried out to knock out VDR expression in the WM164 cell line. The method was used to change the cellular genome specifically to knock out the expression of the *VDR* gene. The cells were plated in 25 cm^2^ flasks and cultured for 24 h, followed by replacing the medium with medium containing polybrene (10 µg/mL) to stabilize lentivirus and incubate with human VDR sgRNA CRISPR All-in-One Lentivirus or scrambled lentivirus (Applied Biological Materials Inc., Richmond, BC, Canada) for the next 24 h. The sgRNA was designed to target all 3 isoforms of VDR (NM_000376, NM_001017535, NM_001017536) (Figure 1A). After incubation, the medium was changed to a medium containing 5 µg/mL puromycin for selection of lentivirus transduced cells. The resulting cells with the *VDR* gene knocked out are designated as WM164 VDR KO, and the cells transduced with an “empty” lentivirus served as a control (WM164 scramble). VDR expression was checked for scramble and VDR KO lines before experiments were performed and further every 4 months after lentivirus treatment to ensure that VDR was not expressed in the WM164 KO.

### 2.4. Western Blot Analysis of VDR Expression

The Western blot method used in the present study has been described previously [52]. VDR (D-6) primary monoclonal anti-mouse antibody (Santa Cruz Biotechnology, Inc., Dallas, TX, USA) was used after a 1:200 dilution with 5% skim milk in TBS-T buffer. m-IgGκ BP-HRP (Santa Cruz Biotechnology, Inc., Dallas, TX, USA) in 5% skim milk (1:5000) was used as secondary antibody. Immuno-reactivity was detected using SuperSignal West Pico Chemiluminescent Substrate (Thermo Fisher Scientific, Waltham, MA, USA). The original Western Blots data was shown in the Supplementary Materials.

### 2.5. MTS Assay of Melanoma Cell Proliferation

Both scramble and VDR KO cell types were plated onto a 96-well plate at a density of 0.5 × 10^3^ cells/well. Cells were incubated with selected vitamin D3 compounds at concentrations from 10^−7^ to 10^−10^ M for period of 24 h. MTS solution (Promega, Madison, WI, USA), 10 µL/well, was then added and after 3 h the absorbance was measured using a Cytation 5 plate reader at 490 nm. Absorbances were analyzed using Gen 5.3 software (BioTek, Winooski, VT, USA).

### 2.6. Cell Counting

The VDR KO and scrambled cells were seeded onto 24-well plates in triplicate, at 1 × 10^4^ cells per well and counted daily using a hemocytometric chamber. The cells were washed with PBS solution and then incubated with 100 μL trypsin 0.25% (Corning, NY, USA) for 5 min. To stop cell trypsinization medium with 10% FBS was added, 400 or 900 μL (depending on the number of cells). A 10 μL aliquot of cells was taken and the cells were counted under a microscope using a Bürker hemocytometer. A second method of checking the proliferation was to measure cell surface area before counting them manually in a hemocytometric chamber. Wells were photographed using a Cytation 5 instrument (BioTek, Winooski, VT, USA) and the covered surface area was analyzed using Gen 5.3 software (BioTek, Winooski, VT, USA).

### 2.7. Analysis of Spheroid Formation in Culture

The WM164 cells were cultured for the formation of spheroids in DMEM containing 20 ng/mL epidermal growth factor, 10 ng/mL basal fibroblast growth factor, 5 µg/mL insulin and 0.4% bovine serum. Cells at a concentration of 5 × 10^3^/mL were added to the medium and incubated with factor B27, growth factor to support the formation of spheroids in cell lines, at a dilution of 1:50 (Gibco, Waltham, MA, USA). The cells were plated onto a 96 well ultralow attachment plate (Costar^®^, Corning, NY, USA) 200 µL/well. The wells at the edge of the plate were filled with PBS to ensure adequate humidity inside the plate. After a week of incubation, the resulting spheroids were counted manually and using a Cytation 5 instrument (BioTek, Winooski, VT, USA). Data were analyzed using Gen 5.3 software (BioTek, Winooski, VT, USA).

### 2.8. Colony Formation Assay

The WM164 cells were seeded at 3 × 10^3^/well, onto a 12-well culture plate (TPP) using DMEM medium containing selected vitamin D3 derivatives (1,25(OH)_2_D3, 25(OH)D3, 1,20(OH)_2_D3, or 20(OH)D3) at concentrations ranging from 10^−7^ to 10^−10^ M. After three days, the medium was changed, and 7 days after seeding the cells were stained with crystal violet and the number of colonies formed was analyzed using Gen 5.3 software (BioTek, Winooski, VT).

### 2.9. Cell Migration Assay

The VDR KO and scrambled cells were seeded at 2 × 10^4^ cells in 70 μL onto 3 special removable silicone wells (ibidi^®^, Gräfelfing, Germany) in a 24-well plate. Cells were incubated in serum-free medium for 24 h. At 95% confluence, the wells were removed, thus creating two scratches in every well, each 5 μm wide. After the formation of scratches, the cells were incubated with selected vitamin D3 derivatives at a concentration of 10^−7^ M, or ethanol as a control. The plate was placed in a Cytation 5 reader at 37 °C and 5% CO_2_ where photos of each well were taken simultaneously every hour for 70 h. Migration analysis was performed using Gen 5.3 software.

After pictures were taken, we defined a rectangle with an area 10 µm^2^ on and around the scratch and counted the area occupied by the cells. The size of the rectangle occupied by cells directly correlated with the scratch that was covered.

### 2.10. Statistical Analysis

Results are presented as the mean ± SEM. Calculations of statistical significance of the tests were carried out with GraphPad Prism 4 (San Diego, CA, USA). Depending on the data, a two-way ANOVA or Student’s *t*-test analysis was performed. Given the exploratory nature of this study, there was no correction made for multiple testing, and statistical significance was set at *p* value < 0.05. Values are *p* < 0.05 *, *p* < 0.01 **, *p* < 0.001 ***, *p* < 0.0001 ****.

## 3. Results

In 2010, Brożyna and co-authors showed that as the malignancy of cancer increases, the expression of the VDR decreases in patients with skin melanoma [31]. To further investigate this relationship, we knocked out the *VDR* gene in WM164 melanoma cells using CRISPR/Cas methodology as described in Materials and Methods. The lack of vitamin D receptor expression was confirmed by Western blotting, which showed that unlike the scramble control, no protein corresponding to the VDR was present in the WM164 KO cells (Figure 1B and Figure S1).

### 3.1. VDR Expression Affects Cell Proliferation, Colony and Spheroid Formation

**Figure 2:**
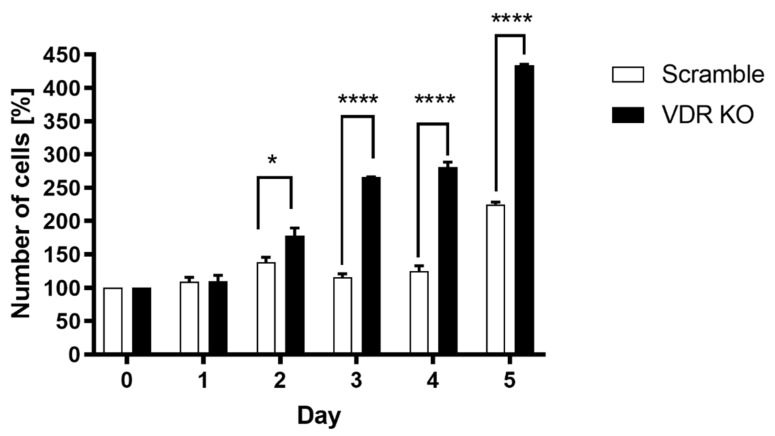
Proliferation rate of VDR KO WM164 cells versus scramble controls. The number of cells (% relative to day 0) was determined each day for 5 days from seeding for both cell types, which were seeded at the same initial concentration. Statistical significance was determined using the *t*-test, *p* < 0.05 *, *p* < 0.0001 ****, *n* = 6.

**Figure 3:**
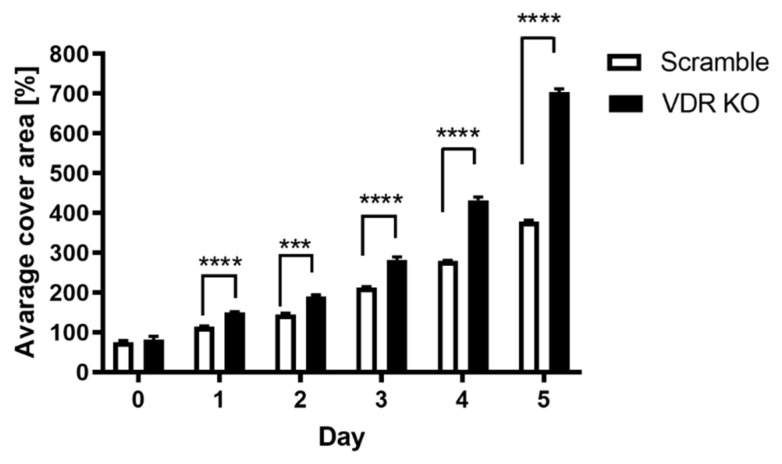
The average surface area covered by scramble and VDR KO WM164 cells over 5 days of growth. Cell surface area was measured for scramble controls and VDR KO cells over 5 days as in Figure 2, with both cell types originally being seeded (day 0) at the same concentration. Statistical significance was determined by *t*-test, *p* < 0.001 ***, *p* < 0.0001 ****, *n* = 6.

After confirming that the VDR was knocked out in WM164 cells, we investigated how VDR expression affects cell proliferation, colony and spheroid formation. Daily cell counting using a hemocytometric chamber and daily measurements of the space occupied by the cells confirmed that knocking out the VDR in WM164 melanoma cells accelerates their multiplication rate. The number of VDR KO cells was significantly higher than for scramble cells from days 2 to 5 of the experiment, being more than double on days 3 and 4 (Figure 2). The average cell surface area for VDR KO cells was also significantly higher than for scramble controls on days 4 and 5 (Figure 3).

**Figure 4:**
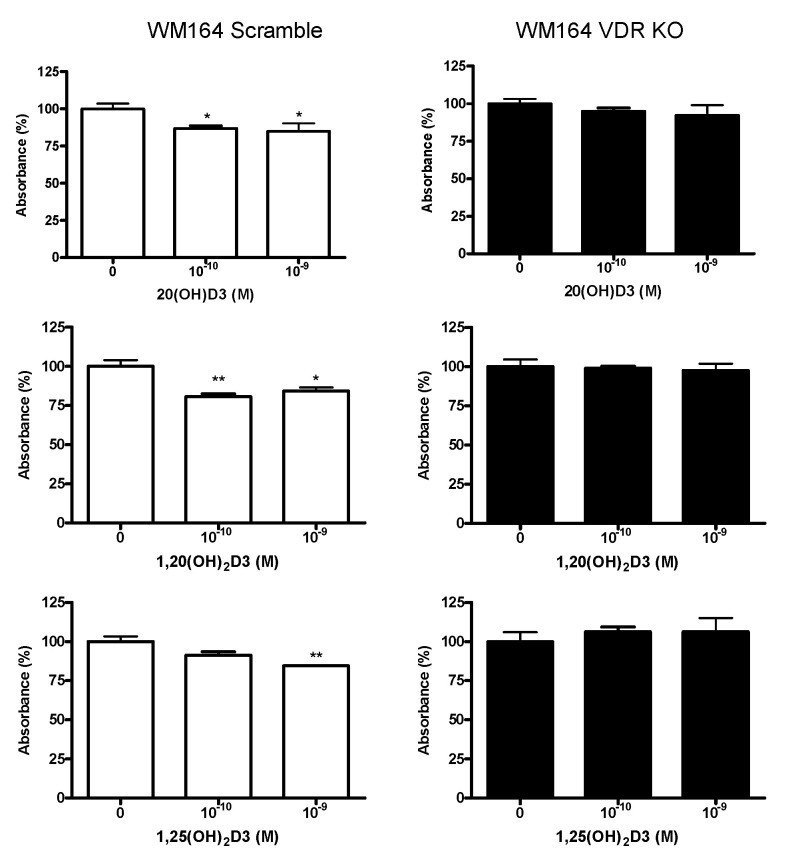
Active forms of vitamin D3 inhibit the proliferation of WM164 cells through the VDR. Both cell types, WM164 scramble and VDR KO cells, were seeded at the same concentration. The MTS assay was performed after 24 h of incubation with vitamin D3 derivatives. Statistical significance was determined by *t*-test, *p* < 0.05 *, *p* < 0.01 **, *n* = 4.

To determine the anti-proliferative effect of vitamin D3 derivatives, scramble and VDR KO cells were incubated for 24 h with 1,25(OH)_2_D3, 1,20(OH)_2_D3 or 20(OH)D3 at concentrations of 10^−9^ and 10^−10^ M. Proliferation was measured using the MTS assay, which measures mitochondrial activity. Vitamin D3 derivatives had a small but significant inhibitory effect on the proliferation of the scramble cell line, typically about 20%, but they did not have a significant effect on the VDR KO cells (Figure 4).

**Figure 5:**
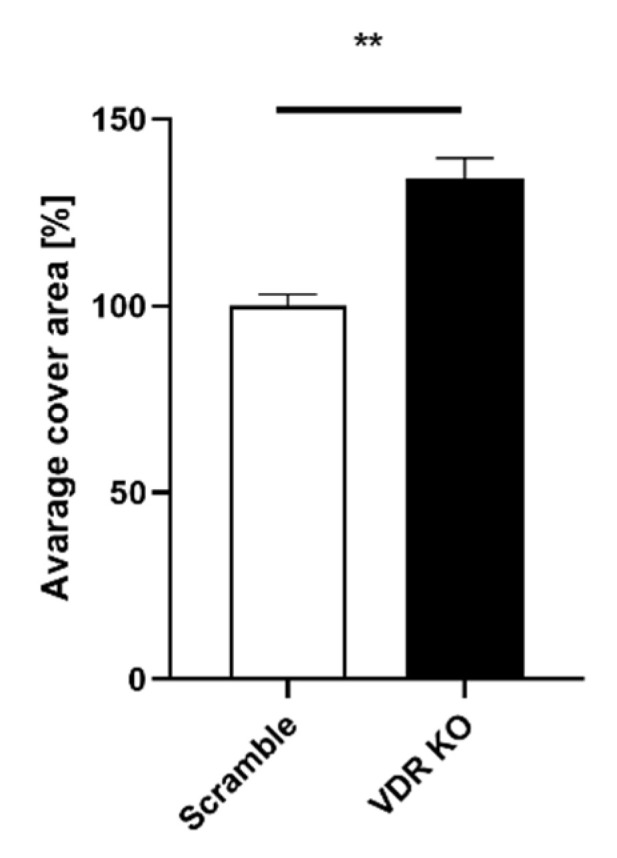
The area occupied by colonies formed after 14 days of incubation of WM164 VDR KO was greater than scramble cells. Cells were seeded at 3 × 10^3^/well and after 14 days were fixed and dyed with crystal blue. The images of wells were made using a Cytation 5 reader with Gen 5.3 software, and % area coverage relative to the scramble control (100%) was determined. Statistical analysis was via *t*-test, *p* < 0.01 **, *n* = 6.

**Figure 6:**
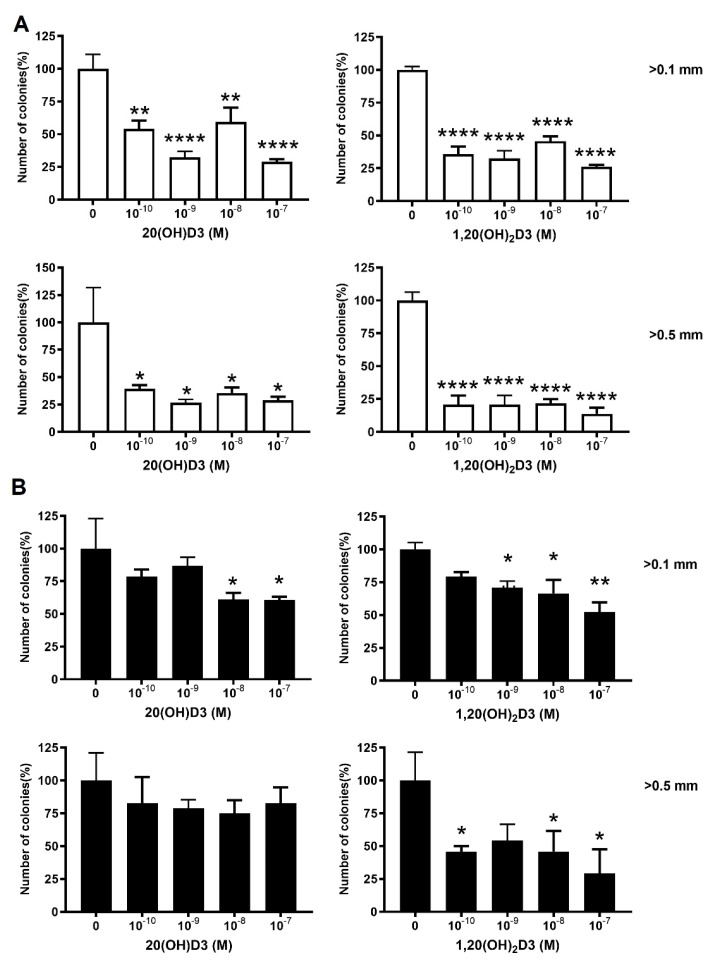
The effect of vitamin D derivatives on the formation of small and large colonies by VDR KO cells and scramble controls. The number of colonies (% of ethanol control) with diameters of 0.1–0.5 and >0.5 mm for scramble (**A**) or WM164 VDR KO (**B**) cells was determined after 7 days of incubation with 1,20(OH)_2_D3 or 20(OH)D3 at the indicated concentrations. One-way ANOVA showed significant differences: *p* < 0.05 *, *p* < 0.01 **, *p* < 0.0001 ****, *n* = 6.

After seeing the effects on cell proliferation, we checked the ability of VDR KO cells to form colonies. The VDR KO produced approximately 40% more colonies than the scramble control (Figure 5). The influence of vitamin D3 derivatives on colony formation by WM164 VDR KO cells compared to scramble controls was also examined (Figure 6). Cells were incubated for 7 days with selected vitamin D3 derivatives (20(OH)D3 and 1,20(OH)_2_D3) at concentrations from 10^−7^ to 10^−10^ M. Both secosteroids significantly decreased colony formation by the scramble melanoma cells at all concentrations tested compared to the ethanol control (Figure 6A), by up to about 75% at the highest concentrations. In contrast, 20(OH)D3 and 1,20(OH)_2_D3 had lesser effects on colony formation by the VDR KO cells (Figure 6B). 20(OH)D3 did not affect the formation of larger colonies (>0.5 mm) and only diminished the formation of smaller colonies (0.2–0.5 mm) at concentrations of 10^−7^ and 10^−8^ M. 1,20(OH)_2_D3 was somewhat more effective with higher doses significantly inhibiting the formation of both small and large colonies.

**Figure 7:**
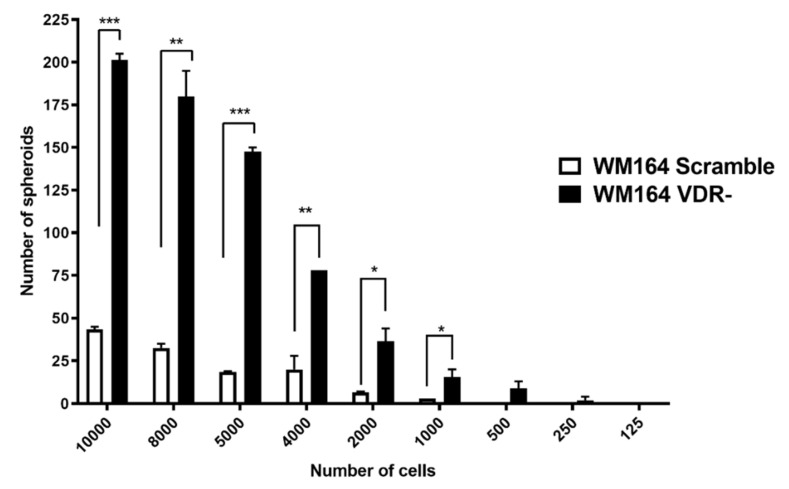
Relationship between the number of seeded cells and spheroid formation for WM164 VDR KO and scramble cells. Cell were seeded in numbers ranging from 125 to 10,000 cells/well, as indicated, and incubated for 7 days. Statistical analysis was conducted using the *t*-test, *p* < 0.05 *, *p* < 0.01 **, *p* < 0.001 ***, *n* = 6.

**Figure 8:**
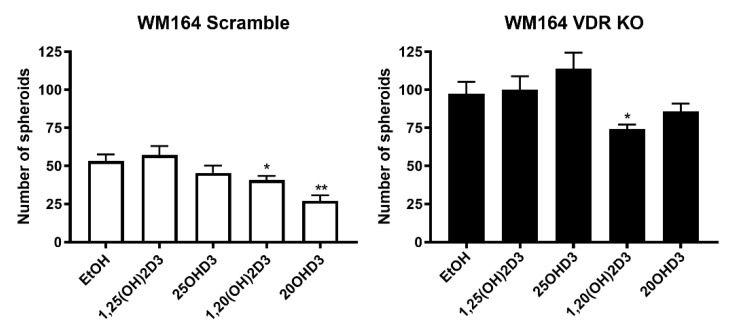
The effect of vitamin D derivatives on spheroid formation by WM164 VDR KO and scramble cells. Both cell types were seeded at 2000 per well and incubated with secosteroids, as indicated, at 10^−7^ M for 7 days. The respective ethanol vehicle controls were used for evaluating statistical differences for the secosteroid treatments. Differences vs. ethanol control were analyzed using the *t*-test, *p* < 0.05 *, *p* < 0.01 **, *n* = 6.

The ability of WM164 VDR KO cells and scramble control cells to form spheroids was used as an indicator of the tumor-forming properties of these cells. Initially we determined the relationship between the number of seeded cells and the number of spheroids formed over a 7-day incubation period. Cells were seeded at numbers ranging between 125 and 10,000. With higher numbers of seeded cells (5000–10,000), significantly more spheroids were formed by the VDR KO cells than the scramble controls (Figure 7). Incubation of VDR KO cells with vitamin D3 derivatives at 10^−7^ M revealed that only 1,20(OH)_2_D3 significantly reduced the number of spheroids formed, whereas a reduction was seen for the WM164 scramble cells with both 20(OH)D3 and 1,20(OH)_2_D3 (Figure 8).

### 3.2. VDR Expression Affects Cell Migration

**Figure 9:**
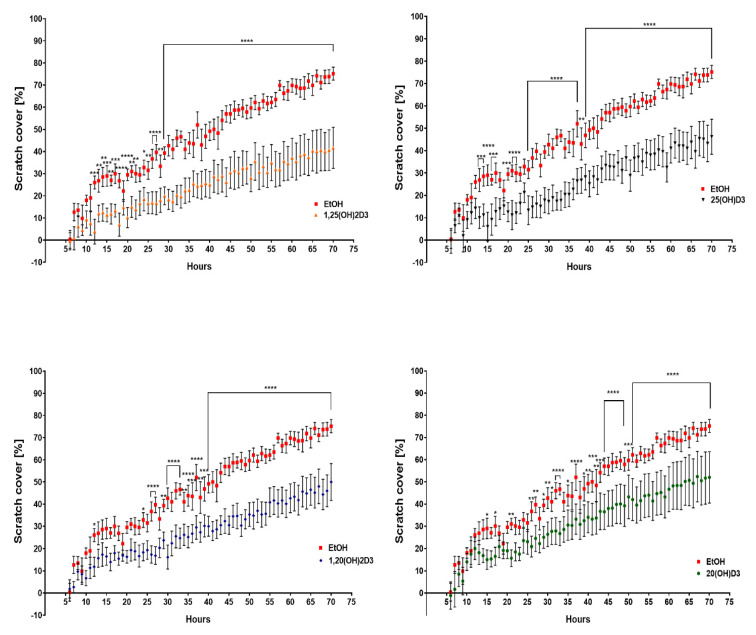
The effect of vitamin D derivatives on the migration of WM164 scramble cells determined with the scratch assay. Movement of cells into the scratch area was observed over 70 h of incubation with secosteroids at 10^−7^ M or the ethanol vehicle as control. Pictures of the scratch area were taken every hour for 70 h using a Cytation 5 instrument and were analyzed using Gen 5.3 software (BioTek, Winooski, VT, USA), two-way ANOVA, *p* < 0.05 *, *p* < 0.01 **, *p* < 0.001 ***, *p* < 0.0001 ****, *n* = 7. The line defines all time points with the same statistical significance.

**Figure 10:**
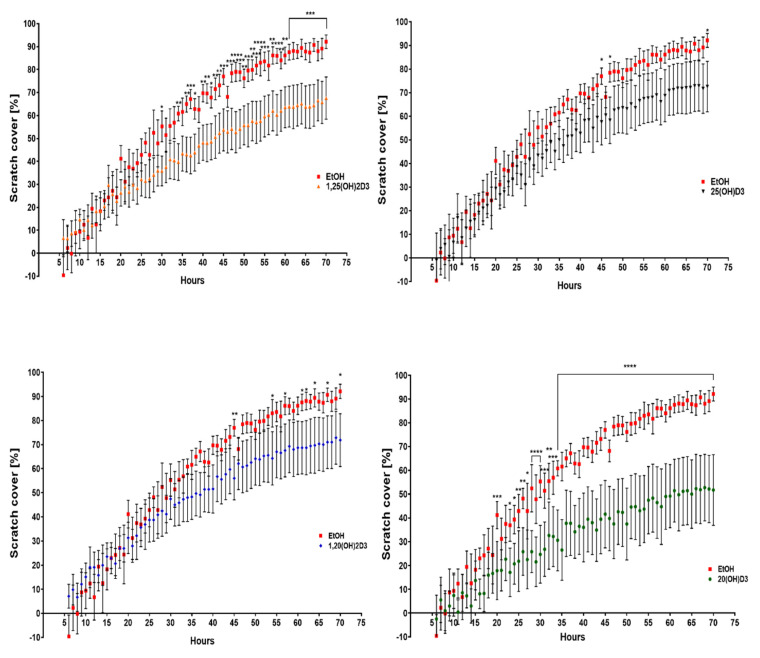
The effect of vitamin D derivatives on the migration of VDR KO type WM164 determined with the scratch assay. Movement of cells into the scratch area was observed over 70 h of incubation with secosteroids at 10^−7^ M or the ethanol vehicle as control. Pictures of the scratch area were taken every hour for 70 h using a Cytation 5 instrument and were analyzed using Gen 5.3 software (BioTek, Winooski, VT, USA), two-way ANOVA, *p* < 0.05 *, *p* < 0.01 **, *p* < 0.001 ***, *p* < 0.0001 ****, *n* = 7. The line defines all time points with the same statistical significance.

To compare the migration abilities of VDR KO and scrambled cells, the extent by which the cells could fill a “scratched” area of the culture well was determined (Figure 9 and Figure 10). Incubation of WM164 scramble cells with the four vitamin D3 derivatives significantly reduced this area to less than 50% by the end of 70 h (Figure 9). After 70 h of incubation with the ethanol vehicle (control), the VDR KO type cells covered 90% of the scratch area, while scramble cells covered only 75% of the area (Figure 10). The incubation of VDR KO cells with vitamin D3 derivatives had a much smaller effect on the coverage of the scratch area.

## 4. Discussion

The VDR is implicated in the regulation of an array of biological activities, including development and progression of cancer, for example breast, prostate and ovarian cancers and melanoma [53,54,55]. Brozyna et al. [31,46] showed that less advanced tumors exhibited significantly higher VDR expression than those in the advanced stage. In order to explore the role of the VDR in melanoma cells in more depth, we established a melanoma cell line with the expression of the VDR knocked out and compared this to control cells with a functional VDR. Our results indicate that VDR expression affects the characteristics of the melanoma line originally derived from human skin. Knocking out of VDR expression in the WM164 human skin melanoma cell line not only caused changes in cell morphology but also accelerated the growth rate, as measured by proliferation and colony formation assays. Furthermore, spheroid formation, as an indicator of the tumor-forming ability of the cells, was more prominent in melanoma cells with the VDR knocked out. This is in line with the results of Muralidhar et al. [35] who reported that there is a correlation between expression of VDR and melanoma progression and antitumor immunity. Their findings revealed that higher expression of VDR was correlated with upregulated pathways mediating antitumor immunity and with downregulated proliferative pathways [35]. Other studies have shown the importance of the VDR for protecting against tumor development, such as development of breast cancer in VDR deficient mice [56] and in response to UV irradiation [57]. When VDR KO mice were exposed to UVR, they showed greater stimulation of carcinogenesis than their wild-type siblings, and tumor proliferation continued to increase for at least 48 h, while tumor proliferation in wild-type mice reached a plateau after 24 h [58]. Similarly, Sertznig et al. [59] showed that the expression of VDR was stronger in the 1,25(OH)_2_D3-sensitive melanoma cells such as MeWo and SK-Mel-28, compared to 1,25(OH)_2_D3-resistant melanoma cell lines such as SK-Mel-5 and SK-Mel-25. They also reported that treatment with 1,25(OH)_2_D3 increased VDR expression in MeWo and SK-Mel-28 cells but not in SK-Mel-5 and SK-Mel-25 cell lines. Similar to the case of breast cancer which has a higher predisposition to growth and the development of metastases [60], our study shows that WM164 VDR KO cells proliferate and migrate faster than the control scramble cells type.

Oda and colleagues supported the above in vivo studies with in vitro ones where the expression of VDR and the DRIP205 coactivator were decreased. They showed that the lack of the VDR was associated with an increase in proliferation and a reduction of the differentiation of human keratinocytes [61]. Another study [48] confirmed that the expression of the vitamin D receptor in breast cancer patients was inversely proportional to tumor aggressiveness, including tumor size. There was also a correlation between the 25(OH)D3 serum concentration and risk of aggressive breast tumor. High VDR expression determined a less malignant phenotype and was associated with better prognosis. The loss of VDR affected tumor melanoma behavior, allowing disease progression, thus making VDR expression a prognostic marker for routine histopathological evaluation [46].

Our previous studies show that novel CYP11A1-derived vitamin D derivatives inhibit proliferation of different cells, including melanoma [38]. In the current study we show that these vitamin D_3_ hydroxyderivatives, 1,20(OH)_2_D3 and 20(OH)D3, inhibit the proliferation of human WM164 melanoma cells, with a similar effect to the already characterized 1,25(OH)_2_D3. This supports that the recently uncovered pathways for the synthesis of these hydroxyderivatives from vitamin D3 represent alternative ways by which vitamin D3 can be activated. We also demonstrate that knocking out VDR expression in human skin melanoma cells increases their proliferation and colony and spheroid formation capacity. This suggests that expression of VDR is connected with the tumor malignancy of human skin melanoma. This opens new possibilities in the methods of diagnosis and treatment of not only skin melanomas but also other cancers.

Significant effects of 20(OH)D3 and 1,20(OH)_2_D3 on the WM164 melanoma cells lacking VDR expression, shown in this study, support recent findings that other receptors besides the VDR mediate some of the effects of vitamin D derivatives [24,62,63,64,65]. Our results support previous observations by Brożyna et al. [31,46,66,67] and Markiewicz et al. [68] that VDR expression in patient melanoma samples can help with the prognosis and choice of the best therapy. These findings are also consistent with several studies that provide evidence for a role of vitamin D signaling in melanoma prevention and attenuation of disease severity [35,36,47,66,69,70,71,72]. It must also be noted that VDR is considered as a tumor suppressor in cutaneous carcinogenesis [58,73,74]. Based on the above as well as on the current data, we propose that the VDR is also a tumor suppressor in melanoma. However, further research into the effects of the non-calcemic 20(OH)D3 and the low calcemic 1,20(OH)_2_D3 on cancer lines is necessary to fully validate their potential as therapeutic agents for melanoma therapy.

## 5. Conclusions

Our studies demonstrating that knocking out VDR expression in human melanoma cells increases parameters of malignancy indicate that expression of VDR is connected with an increased malignant behavior in melanoma cells. This is consistent with clinicopathological studies showing an inverse correlation between melanoma progression and VDR expression, with very poor disease outcome in VDR negative melanomas. Therefore, we propose that VDR can act as a melanoma tumor suppressor gene.

Classical (1,25(OH)_2_D3) and CYP11-derived (20(OH)D3, 1,20(OH)_2_D3) hydroxyderivatives of vitamin D inhibited cell proliferation, migration rate and the ability to form colonies and spheroids in melanoma cells. Silencing the VDR attenuated these actions, but not completely. Thus, vitamin D3 hydroxyderivatives are good candidates for melanoma therapy with their main mechanism of action involving VDR; however, action on other nuclear receptors cannot be excluded and remains to be investigated. These findings form a basis for future preclinical studies on the efficacy of CYP11A1-derivatives against human melanomas.

## Data Availability

The data presented in this study are available on reasonable request from the corresponding author.

## Supplementary Materials

The following are available online at https://www.mdpi.com/article/10.3390/cancers13133111/s1, Figure S1: Western blot analysis for VDR and β-actin protein expressions in scrambled and VDR knockout WM-164 melanoma cells.
