# Supplementary material for: Knocking out the Vitamin D Receptor Enhances Malignancy and Decreases Responsiveness to Vitamin D3 Hydroxyderivatives in Human Melanoma Cells
Source: Cancers (Basel). 2021 Jun 22;13(13):3111. doi: 10.3390/cancers13133111 (PMC8269360; doi:10.3390/cancers13133111)
Supplement: Supplementary file 1 [file cancers-13-03111-s001.zip › cancers-1238975-supplementary.pdf]

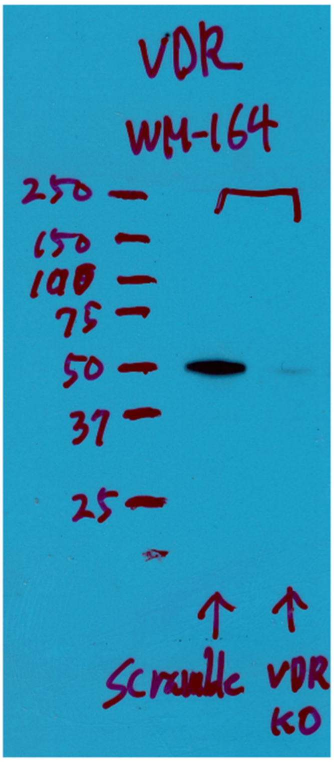

VDR

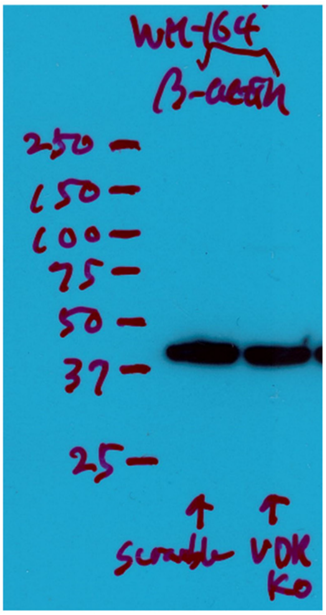

β-actin

Supplementary Figure 1. Western blot analysis for VDR and β-actin protein expressions in scrambled and VDR knockout WM-164 melanoma cells. For details see materials and methods
